# The immunosenescence-related factor DOCK11 is involved in secondary immune responses of B cells

**DOI:** 10.1186/s12979-021-00259-4

**Published:** 2022-01-03

**Authors:** Yuma Sugiyama, Mitsuhiro Fujiwara, Akihiko Sakamoto, Hiromichi Tsushima, Akihiko Nishikimi, Mitsuo Maruyama

**Affiliations:** 1 Department of Inflammation and Immunosenescence, Geroscience Research Center, Research Institute, National Center for Geriatrics and Gerontology, 7-430 Morioka-cho, Obu, Aichi 474-8511 Japan; 2 Biosafety Division, Research Institute, National Center for Geriatrics and Gerontology, Obu, Aichi Japan; 3 Department of Aging Research, Nagoya University Graduate School of Medicine, Nagoya, Japan

**Keywords:** Dock11, Antibody-producing cells, B-lymphocytes, Secondary immune response

## Abstract

**Background:**

Memory B cells are an antigen-experienced B-cell population with the ability to rapidly differentiate into antibody-producing cells by recall responses. We recently found that dedicator of cytokinesis 11 (DOCK11) contributes to the expansion of antigen-specific populations among germinal center B cells upon immunization. In comparison, limited information is available on the contribution of DOCK11 to secondary humoral immune responses.

**Results:**

In this study, effects of the DOCK11 deficiency in B cells were examined on secondary immune responses to protein antigen. The lack of DOCK11 in B cells resulted in the impaired induction of antibody-producing cells upon secondary immunization with protein antigen. DOCK11 was dispensable for the recall responses of antigen-experienced B cells, as demonstrated by the comparable induction of antibody-producing cells in mice given transfer of antigen-experienced B cells with no DOCK11 expression. Instead, the lack of DOCK11 in B cells resulted in the impaired secondary immune responses in a B cell-extrinsic manner, which was recovered by the adoptive transfer of cognate T cells.

**Conclusions:**

We addressed that intrinsic and extrinsic effects of DOCK11 expression in B cells may contribute to secondary humoral immune responses in manner of the induction of cognate T-cell help.

**Supplementary Information:**

The online version contains supplementary material available at 10.1186/s12979-021-00259-4.

## Background

Immunological memory is a hallmark of the immune system that provides rapid responses to secondary pathogenic challenges. Memory B cells are an antigen-experienced B-cell population with the ability to rapidly differentiate into antibody-producing cells by recall response [1–4]. Memory B cells, as well as plasma cells are mainly generated from the germinal center (GC), where antigen-primed B cells undergo intense proliferation in association with follicular helper T (T_FH_) cells and follicular dendritic cells (FDC). Within GCs, B cells undergo several processes such as somatic hypermutation, which alters V region of immunoglobulin genes to be matured antigen receptors with high affinity against antigen. B cells with improved affinity for the antigen receive survival signals from antigen and T_FH_ cells. Consequently, B cells producing more effective antibodies are selected and differentiate into memory B and plasma cells as terminally differentiated B cells. Although recent studies have provided detailed insights into the mechanisms that determine B cell fate in GC, how memory B cells are generated has still not to be fully unveiled.

Like other immune functions, immunological memory formation against new antigens is impaired with age, which results in limited responses to vaccination in the elderly. Immunohistochemistry and flow cytometry analysis showed size and number of GCs reduced with age [5, 6]. Due to the defect in GC formation, antibody affinity maturation and subsequent B cell differentiation are compromised in aged individuals. The impairment of GC reaction seems to be due to the dysfunction of multiple immune cells including B cells, T_FH_ cells, and FDCs [7]. For a better understanding of the age-related defects in GC reactions and immunological memory formation, the molecular function of age-associated factors involved in GC reaction should be investigated. We previously isolated a guanine nucleotide exchange factor called dedicator of cytokinesis 11 (DOCK11, also known as Zizimin2) is a member of CDM (Ced-5, DOCK180, and Myoblast City) family guanine nucleotide exchange factor mainly expressed in immune cells [8, 9]. DOCK11 catalyzes the GDP-GTP exchange reaction of Cdc42 through its DHR-2 (also known as CZH-2) domain located in the C-terminus region. We also revealed that DOCK11 overexpression induced Cdc42-mediated reconstitution of actin cytoskeletons resulting in filopodial formation [9]. We successively demonstrated that DOCK11 was associated with bone marrow B cell development and marginal zone B cell formation [10]. In addition to this function, we recently also found that DOCK11 potentially contributes to the expansion of antigen-specific populations among GC B cells upon immunization [11]. Mechanistically, the lack of DOCK11 resulted in the attenuation of B cell-intrinsic signaling. The expression of DOCK11 in the spleen of aged mice (24 months old) decreased compared with that in young mice (7–8 weeks old) by approximately 50%, suggesting expression of DOCK11 is regulated by age-dependent mechanism [9]. To clarify the influence of age-associated DOCK11 downregulation, we investigated the impact of a DOCK11 deficiency in B cells on the secondary immune responses. We found that the specific antibody production and antiviral immunity upon secondary challenge were impaired in DOCK11 conditional knockout mice in B cells. Adoptive transfer experiments revealed that DOCK11 deficiency did not affect the function of the antigen-specific B cells transferred into wild type mice. However, in contrast, antibody production upon secondary immunization was impaired when the conditional knockout mice were used as recipients, indicating DOCK11 expression by B cells may contribute to the recall reaction by B cell-extrinsic manner.

## Results

### Impact of the DOCK11 deficiency in B cells on secondary immune responses

**Fig. 1:**
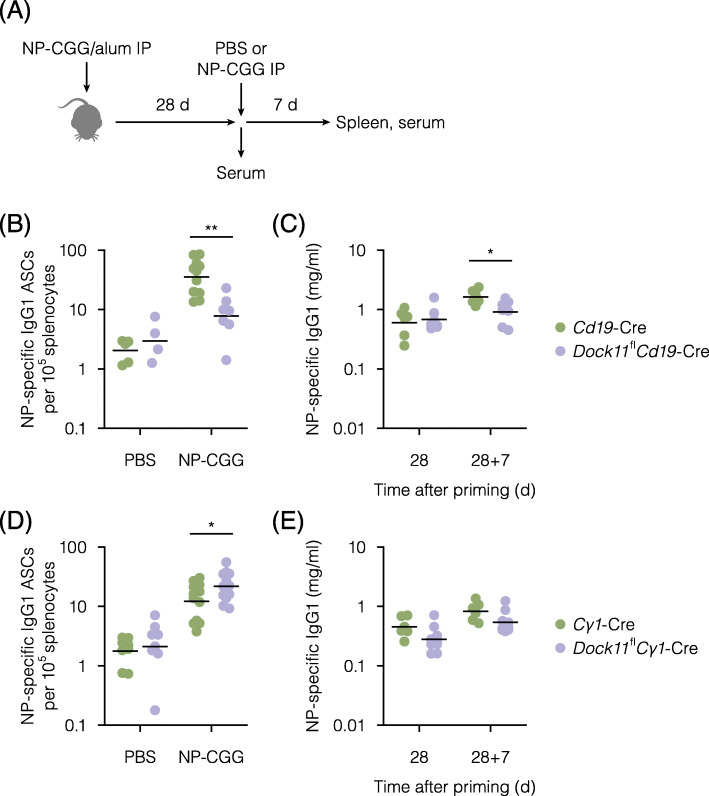
Impact of the DOCK11 deficiency in B cells on secondary immune responses. **A**, Experimental outline to examine secondary immune responses. **B** and **D**, Numbers of NP-specific IgG1^+^ antibody-secreting cells (ASCs) in the spleen from the indicated strains immunized with alum-precipitated NP-CGG, followed by boost immunization or not (PBS) with NP-CGG 28 d later, as measured by an ELISPOT assay 7 d after boost immunization. Each point represents an individual mouse. Bars represent geometric means. Data are pooled from three independent experiments, using four or more mice per experimental group. The values of anti-NP IgG1^+^ASC/10^5^ splenic cells ± SE in the control group and the experimental group in B are 2.0 ± 0.4 and 3.7 ± 1.4 (without immunization) and 43.2 ± 7.9 and 10.0 ± 2.6 (with immunization), respectively. The values of anti-NP IgG1^+^ASC/10^5^ splenic cells ± SE in the control group and the experimental group in D are 2.0 ± 0.3 and 3.0 ± 0.7 (without boosting) and 15.1 ± 2.6 and 25.0 ± 4.0 (with boosting), respectively. **C** and **E**, Concentrations of NP-specific IgG1 in the serum from the indicated strains 28 d post-immunization with alum-precipitated NP-CGG, followed by boost immunization with NP-CGG 7 d later, as measured by an ELISA. Each point represents an individual mouse. Bars represent geometric means. The numbers of mice in each group (B ~ E) are pooled from up to 4 independent experiments, using 4 ~ 8 mice per control group and 6 ~ 12 mice per experimental group. **P < 0.01, *P < 0.05 (Welch’s *t*-test)

To examine the impact of the DOCK11 deficiency in B cells on the secondary immune responses, *Dock11*^fl^ mice [10] were crossed with *Cd19*-Cre mice [12]. The resultant mice would lack the expression of DOCK11 in B cells. *Cd19*-Cre mice were used as a control. After immunization with alum-precipitated NP-CGG, recall responses were induced by an injection of NP-CGG into these mice (Fig. 1A). As a control without recall responses, buffer alone was injected instead of NP-CGG. Antibody-producing cells were enumerated by an ELISPOT assay. In the spleen of *Cd19*-Cre mice, numbers of NP-specific IgG1-producing cells were increased by 17.6 times 7 d after secondary immunization (Fig. 1B). By contrast, only 2.6 times the number of NP-specific IgG1-producing cells were induced by secondary immunization of DOCK11-deficient mice. Correspondingly, serum levels of NP-specific IgG1 were lower in DOCK11-deficient mice as compared with those in *Cd19*-Cre mice after secondary immunization (Fig. 1C). Thus, the lack of DOCK11 in B cells resulted in the impaired induction of antibody-producing cells by secondary immunization.

Recall responses were then examined in mice lacking DOCK11 after the B-cell activation stage. *Cγ1*-Cre mice are a strain in which the expression of Cre recombinase is induced by the transcription of the IgG1 constant region [13]. By crossing *Dock11*^fl^ mice with *Cγ1*-Cre mice, DOCK11 expression would be lost in antigen-experienced B cells. *Cγ1*-Cre mice were used as a control. After immunization with alum-precipitated NP-CGG, recall responses were induced by an injection of NP-CGG into these mice (Fig. 1A). In the spleen of *Cγ1*-Cre mice, numbers of NP-specific IgG1-producing cells were increased by 6.9 times 7 d after secondary immunization (Fig. 1D). Similarly, 10.3 times the number of NP-specific IgG1-producing cells were induced in mice lacking DOCK11 by *Cγ1*-Cre recombinase. Additionally, serum levels of NP-specific IgG1 were similar among these strains (Fig. 1E). Thus, antibody-producing cells seemed to be normally induced by secondary immunization in mice lacking DOCK11 by *Cγ1*-Cre recombinase.

### B cell-intrinsic impact of the DOCK11 deficiency on recall responses

**Fig. 2:**
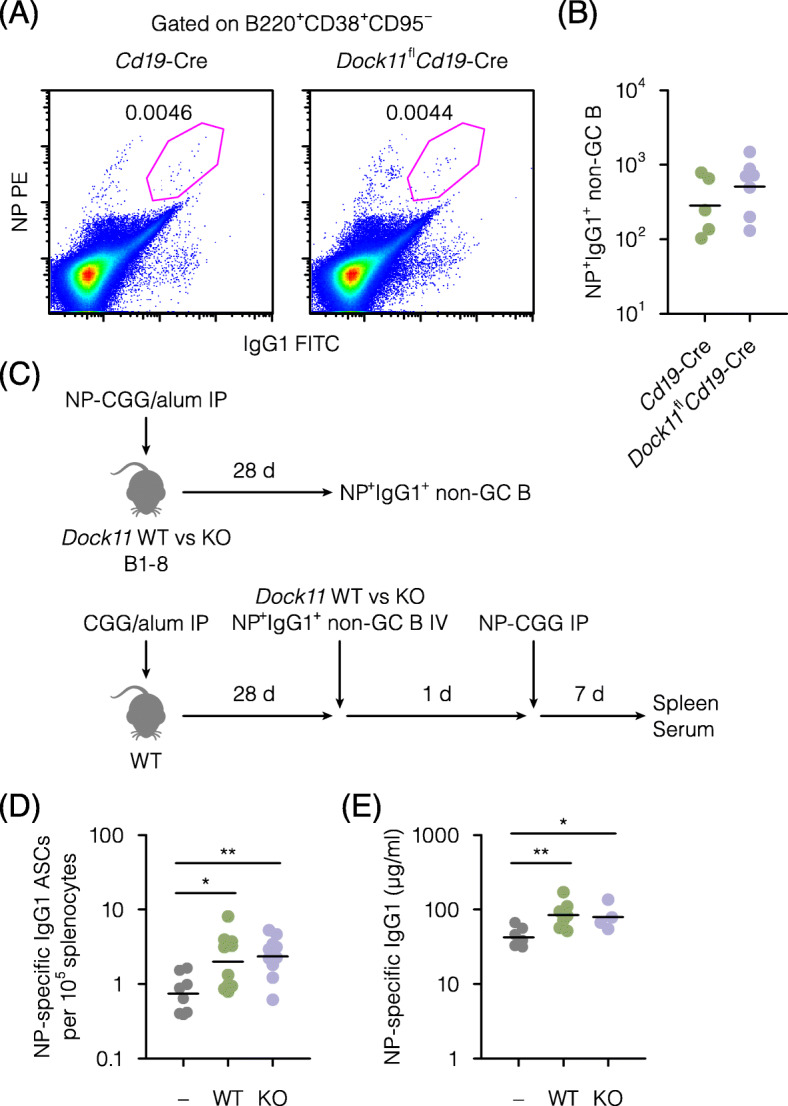
B cell-intrinsic impact of the DOCK11 deficiency on recall responses. **A**, Comparable formation of NP-specific IgG1^+^ non-GC B cells (B220^+^CD38^+^CD95^−^NP^+^IgG1^+^) in the spleen of the indicated strains 28 d post-immunization with alum-precipitated NP-CGG, as measured by flow cytometry. Representative pseudo-color plots are gated on B220^+^CD38^+^CD95^−^ cells. Numbers show percentages of cells in each gate. Detailed gating strategies are shown in Fig. S1. **B**, Numbers of NP-specific IgG1^+^ non-GC B cells in (**A**). Each point represents an individual mouse. Bars represent geometric means. Data are pooled from two independent experiments, using five or more mice per experimental group. **C**, Experimental outline to examine the impact of the DOCK11 deficiency on recall responses of antigen-experienced IgG1^+^ non-GC B cells. NP-specific IgG1^+^ non-GC B cells were isolated from spleens of B1–8 IgH-carrying mice (WT) or DOCK11-deficient counterparts (KO) 28 d post-immunization with alum-precipitated NP-CGG. These cells were then transferred into congenic C57BL/6 recipients (WT) immunized with alum-precipitated CGG 28 d before, followed by secondary immunization with NP-CGG and subsequent analyses 7 d later. **D**, Numbers of NP-specific IgG1 antibody-secreting cells (ASCs) as measured by an ELISPOT assay in the spleen of recipients given transfer or not (−) of DOCK11-sufficient (WT) or deficient (KO) NP-specific IgG1^+^ non-GC B cells, followed by secondary immunization with NP-CGG. Each point represents an individual recipient. Bars represent geometric means. Data are pooled from three independent experiments, using eight or more recipients per experimental group. The values of anti-NP IgG1^+^ASC/10^5^ splenic cells ± SE are 0.9 ± 0.2 (−), 2.7 ± 0.7 (WT) and 2.7 ± 0.4 (KO). **E**, Serum levels of NP-specific IgG1 in (**D**), as measured by an ELISA. **P < 0.01, *P < 0.05 (Tukey’s test)

Upon secondary immunization or infection, antibody-producing cells are induced by the differentiation of memory B cells. Since memory B cells are usually included in NP-specific IgG1^+^ non-GC B cells after immunization with alum-precipitated NP-CGG [14], numbers of this population were enumerated by flow cytometry (Fig. S1). Irrespective of the DOCK11 expression by B cells, NP-specific IgG1^+^ non-GC B cells were normally formed (Fig. 2A, B). Thus, DOCK11 seemed to be dispensable for the formation of these antigen-experienced B cells.

Then, the effects of the DOCK11 deficiency on the recall responses of this population were examined in a cell transfer system (Fig. 2C). As a source of NP-specific IgG1^+^ non-GC B cells, B1–8 IgH-carrying mice [15] were used. The DOCK11-deficient counterpart was obtained by crossing B1–8 IgH-carrying mice with *Dock11* KO mice. After immunization of these mice with alum-precipitated NP-CGG, NP-specific IgG1^+^ non-GC B cells were isolated from spleens by cell sorting (Fig. S2). These cells were then injected into CGG-primed WT recipients (Fig. 2C). After boost immunization with NP-CGG, antibody-producing cells were enumerated by an ELISPOT assay. The potency of NP-specific IgG1^+^ non-GC B cells as memory B cells was confirmed by 2.7 times larger numbers of NP-specific IgG1-producing cells as compared with recipients without adoptive transfer (Fig. 2D). Similarly, 3.2 times larger numbers of NP-specific IgG1-producing cells were formed in recipients given transfer with DOCK11-deficient NP-specific IgG1^+^ non-GC B cells. Correspondingly, serum levels of NP-specific IgG1 were elevated by the adoptive transfer of NP-specific IgG1^+^ non-GC B cells, irrespective of the DOCK11 expression (Fig. 2E). Thus, DOCK11 seemed to be dispensable for the recall responses of these antigen-experienced B cells.

### B cell-extrinsic impact of the DOCK11 deficiency on recall responses

**Fig. 3:**
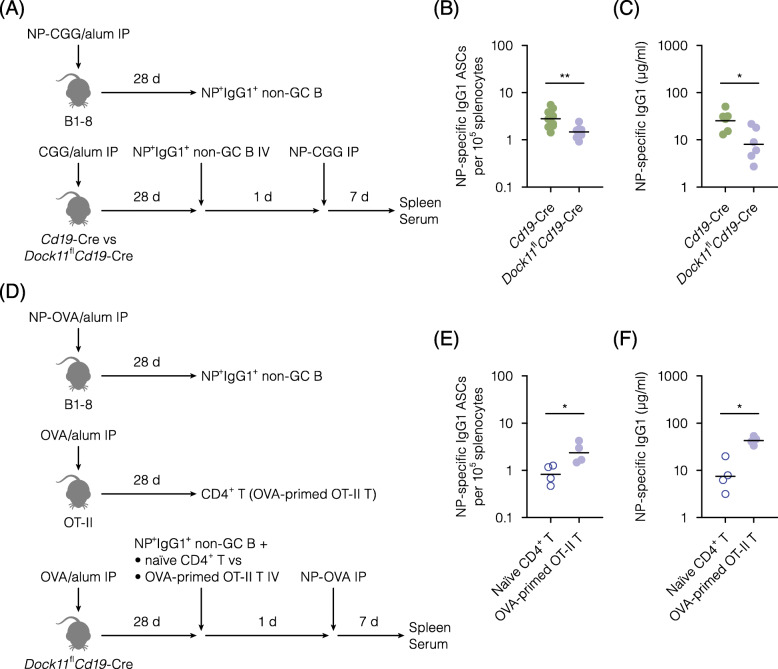
B cell-extrinsic impact of the DOCK11 deficiency on recall responses. **A**, Experimental outline to examine whether the lack of DOCK11 in B cells affects the secondary immune responses in a B cell-extrinsic manner. NP-specific IgG1^+^ non-GC B cells were isolated from spleens of B1–8 IgH-carrying mice 28 d post-immunization with alum-precipitated NP-CGG. These cells were transferred into congenic *Cd19*-Cre mice or DOCK11-deficient counterparts immunized with alum-precipitated CGG 28 d before, followed by secondary immunization with NP-CGG and subsequent analyses 7 d later. **B**, Numbers of NP-specific IgG1 antibody-secreting cells (ASCs) as measured by an ELISPOT assay in the spleen of the indicated recipients given transfer of NP-specific IgG1^+^ non-GC B cells, followed by secondary immunization with NP-CGG. Each point represents an individual recipient. Bars represent geometric means. Data are pooled from three independent experiments, using five or more recipients per experimental group. The values of anti-NP IgG1^+^ASC/10^5^ splenic cells ± SE in Cd19-Cre control group and Dock11^fl^Cd19-Cre group are 2.8 ± 0.5 and 1.6 ± 0.2, respectively. **C**, Serum levels of NP-specific IgG1 in (**B**), as measured by an ELISA. **D**, Experimental outline to examine whether the impaired recall responses of antigen-experienced B cells in the DOCK11-deficient recipients would be recovered by adoptive transfer of cognate CD4^+^ T cells. NP-specific IgG1^+^ non-GC B cells were isolated from spleens of B1–8 IgH-carrying mice 28 d post-immunization with alum-precipitated NP-OVA. CD4^+^ T cells were isolated from spleens of naïve C57BL/6 mice or OT-II mice 28 d post-immunization with alum-precipitated OVA. NP-specific IgG1^+^ non-GC B cells were transferred in combinations with either naïve CD4^+^ T cells or OVA-primed OT-II T cells into congenic *Dock11*^fl^*Cd19*-Cre mice immunized with alum-precipitated OVA 28 d before, followed by secondary immunization with NP-OVA and subsequent analyses 7 d later. **E**, Numbers of NP-specific IgG1 ASCs as measured by an ELISPOT assay in the spleen of the recipients given transfer of NP-specific IgG1^+^ non-GC B cells and the indicated CD4^+^ T cells. Each point represents an individual recipient. Bars represent geometric means. Data are pooled from two independent experiments, using four recipients per experimental group. The values of anti-NP IgG1^+^ASC/10^5^ splenic cells ± SE in naïve CD4^+^T control group and OVA-primed OT-II T group are 0.9 ± 0.2 and 2.6 ± 0.6, respectively. F, Serum levels of NP-specific IgG1 in (**E**), as measured by an ELISA. **P < 0.01, *P < 0.05 (Welch’s *t*-test)

Although DOCK11 expression by B cells was involved in the induction of antibody-producing cells in secondary immune responses, antigen-experienced B cells did not seem to be engaged in the underlying mechanisms. Recent studies have demonstrated that cognate T-cell help contributes to the recall responses of memory B cells [16–19]. Therefore, we next examined whether the lack of DOCK11 in B cells may affect the secondary immune responses in a B cell-extrinsic manner. NP-specific IgG1^+^ non-GC B cells were isolated from B1–8 IgH-carrying mice after immunization with alum-precipitated NP-CGG (Fig. 3A). This population was injected into CGG-primed *Cd19*-Cre mice or DOCK11-deficient counterparts. After boost immunization with NP-CGG, antibody-producing cells were enumerated in these recipients by an ELISPOT assay. The numbers of NP-specific IgG1-producing cells were decreased in DOCK11-deficient recipients (Fig. 3B). Correspondingly, serum levels of NP-specific IgG1 were lower in DOCK11-deficient recipients than in the DOCK11-sufficient control (Fig. 3C). Thus, DOCK11 expression by B cells seemed to be required for the recall responses of the transferred antigen-experienced B cells.

Then, contributions of the T-cell help were examined in a cell transfer system (Fig. 3D). NP-specific IgG1^+^ non-GC B cells were isolated from B1–8 IgH-carrying mice after immunization with alum-precipitated NP-OVA. OT-II mice [20] were used as a source of cognate T cells. After immunization with alum-precipitated OVA, CD4^+^ T cells were isolated from these mice. Naïve CD4^+^ T cells as a control were isolated from naïve WT mice. Then, OVA-primed recipients lacking DOCK11 in B cells were transferred with the NP-specific IgG1^+^ non-GC B cells in combination with either naïve CD4^+^ T cells or OVA-primed OT-II T cells. After boost immunization with NP-OVA, antibody-producing cells were enumerated by an ELISPOT assay. Numbers of NP-specific IgG1-producing cells were recovered by the transfer of OVA-primed OT-II T cells (Fig. 3E). Correspondingly, serum levels of NP-specific IgG1 were also recovered by the transfer of OVA-primed OT-II T cells (Fig. 3F). Thus, impaired recall responses of the antigen-experienced B cells in DOCK11-deficient recipients were recovered by the adoptive transfer of cognate T cells.

### B cell-intrinsic effect of the DOCK11 deficiency in cytokine production

**Fig. 4:**
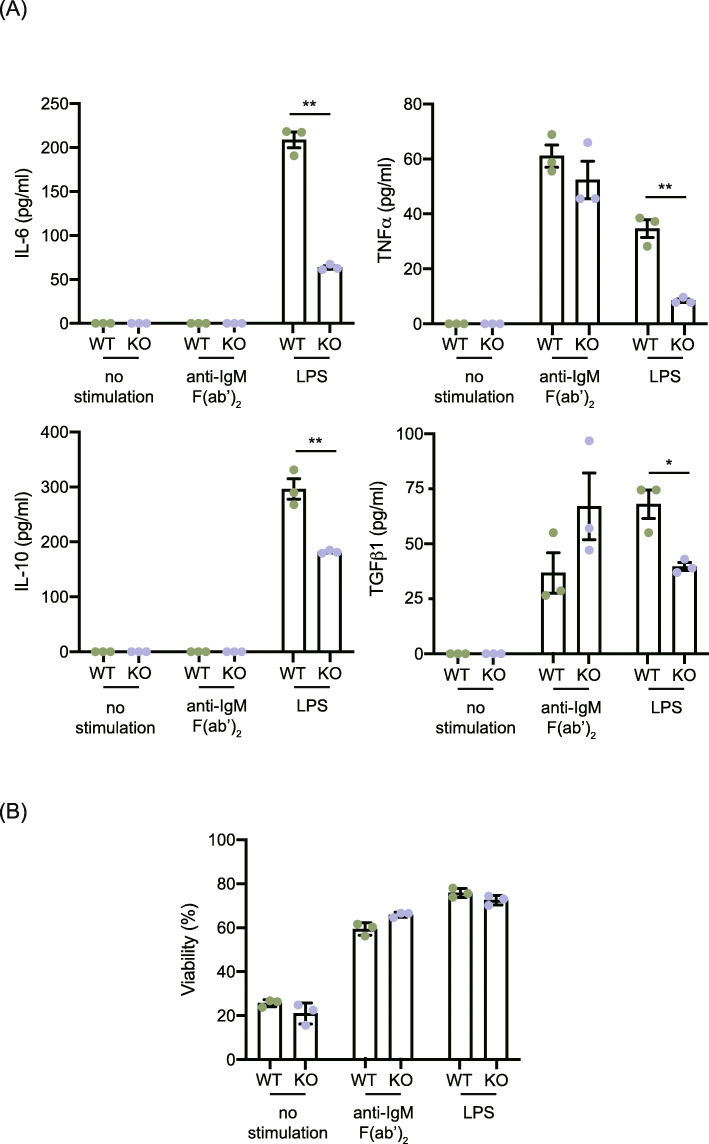
Impact of the DOCK11 deficiency in cytokine production of B cells. **A**, The levels of IL-6, TNFα, TGFβ, and IL-10 in cell culture supernatant were compared between WT and Dock11-deficient B cells 72 h after stimulation of 20 μg/ml anti-IgM F (ab’)_2_ antibody or 10 μg/ml LPS as well as no stimulation. **B**, Viability of each column was calculated by dividing the number of propidium iodide (PI)-negative cells by that of total cells after staining the cells with 2 μg/ml of PI and counted 1 × 10^4^ cells with flow cytometry. Data are mean ± SEM of triplicate wells. **P < 0.01, *P < 0.05 (two-tailed unpaired Student’s t-test)

Finally, we investigated the mechanism whereby Dock11 expression in B cells may ameliorate cognate T cell help in secondary immune responses through in vitro cytokine production assays with B220^+^ Splenic B cells. We isolated the splenic B cell by CD45R MACS beads from DOCK11-deficient mice or WT mice. Then the levels of various cytokines in their supernatants including potent mediators of inflammatory or immune functions, TNFα, IL-6, or TGFβ as well as an anti-inflammatory cytokine, IL-10 were measured after LPS or anti-IgM F (ab’)_2_ stimulation for 72 h in vitro (Fig. 4A).

Interestingly, secretions of IL-6, TNFα, TGFβ and IL-10 were significantly less efficient in DOCK11-deficient mice compared with WT after LPS stimulation (Fig. 4A). However, the result of TNFα and TGFβ after anti-IgM F (ab’) _2_ stimulation could not represent such difference due to DOCK11 deficiency (Fig. 4A). Viability in mock treated B cells were confirmed in vitro culture, consequently there are no significant difference between WT and DOCK11-deficient mice, therefore no specific cytokine production due to DOCK11 deficiency (Fig. 4B). Taken together, this result potentially suggested that the B cell-intrinsic effect of the DOCK11 deficiency is involved in the signaling pathways shared with innate immune function but with B cell receptor signaling, although further experiments remain to be confirmed in detail.

## Discussion

In the present study, we examined the impacts of the DOCK11 deficiency in B cells focused on secondary immune responses. The lack of DOCK11 in B cells resulted in the impaired induction of antibody-producing cells upon the secondary immunization with protein antigen. DOCK11 was dispensable for the recall responses of antigen-experienced B cells, as demonstrated by the comparable induction of antibody-producing cells in mice given transfer with antigen-experienced DOCK11-deficient B cells. Furthermore, interestingly, we found the lack of DOCK11 in B cells indicated the impaired the secondary immune responses in a B cell-extrinsic manner, which was recovered by the adoptive transfer of cognate T cells. Therefore, it is possible that DOCK11 expression in B cells presumably contributes to secondary humoral immune responses through the induction of cognate T-cell help.

We also demonstrated that antibody-producing cells seemed to be normally induced by the secondary immunization in mice lacking DOCK11 by *Cγ1*-Cre recombinase. In these mice, DOCK11 expression would be deleted in antigen-experienced B cells after primary immune response and following class-switching. Therefore, secondary humoral immune responses of these mice are elicited by antigen-experienced B cells without DOCK11 expression. Furthermore, we revealed that DOCK11 may be dispensable for recall responses of antigen-experienced B cells in normal induction of antibody-producing cells as confirmed in a cell transfer system.

In this report, we have shown DOCK11 expression in B cells seemed to be required for the induction of T-cell help in the secondary immune responses. Consistently, we also described that adoptive transfer of cognate T cells recovered the impaired recall responses of antigen-experienced B cells in DOCK11-deficient mice. We have recently demonstrated that the lack of DOCK11 in B cells is involved in the impaired induction of T follicular helper, T_FH_ cells upon immunization [11]. These T cells are an interesting and indispensable population since it has been known that these cells were found to provide cognate help in recall responses of memory B cells [16]. To gain further insight into the function of DOCK11 in the secondary immune response, we analyzed the cytokine production secreted from B cells in DOCK11 deficient mice in vitro and we found that IL-6, TNFα, TGFβ and IL-10 secretion were significantly decreased after not anti-IgM F (ab’)_2_ but LPS stimulation (Fig. 4A). This decline of cytokine secretion in DOCK11 deficient mice B cells may explain and evoke the possibility that DOCK11 plays a role in innate immune signaling response. In support of this possibility, we have preliminary results that we could not detect the secretion of IL-21, IL-4, IL-12p70, IL-17A, IL-13, IFN-γ, BAFF, BCMA, and sCD40L is not detected in DOCK11-deficient B cells but also in B cells from WT in response to both LPS and anti-IgM F (ab’) _2_ stimuli. Taken together, DOCK11 expression by B cells may contribute to the recall responses of antigen-experienced B cells through the induction of memory T follicular helper cells [21].

In conclusion, we examined the impacts of the DOCK11 deficiency in B cells on secondary immune responses. DOCK11 expression by B cells may contribute to secondary humoral immune responses through the induction of cognate T-cell help.

## Conclusions

We addressed that intrinsic and extrinsic effects of DOCK11 expression in B cells may contribute to secondary humoral immune responses in a manner of the induction of cognate T-cell help.

## Methods

### Mice

All mice were maintained on a C57BL/6 background under specific pathogen-free conditions. *Dock11* knockout (KO) [10], *Cd19*-Cre [12], immunoglobulin G1 (IgG1) constant region (*Cγ1*)-Cre [13], *Dock11* flox (*Dock11*^fl^) [10], B1–8f [15], and OT-II mice [20] were described previously. CD45.1 mice were obtained from Sankyo Labo Service. Expression of the OT-II transgene was determined by flow cytometry. Other genotypes were determined by polymerase chain reactions. All animal experiments were performed using 2- to 4-month-old male and female mice with the approval of the institutional review board at the National Center for Geriatrics and Gerontology.

### Immunization

Primary Immunization was performed by an intraperitoneal (IP) injection of 100 μg of alum-precipitated chicken gamma globulin (CGG) (Calbiochem, currently Sigma-Aldrich), ovalbumin (OVA) (Sigma-Aldrich), or these proteins conjugated with 4-hydroxy-3-nitrophenylacetyl (NP) (NP_31–33_-CGG or NP_17_-OVA) (LGC Biosearch Technologies). Secondary immunization was performed by an IP injection of 50 μg of NP_31–33_-CGG or NP_17_-OVA.

### Enzyme-linked immunospot (ELISPOT) assay

For ELISPOT assays, 96-well filter plates (Merck) were coated with 2 μg/ml NP-conjugated bovine serum albumin (NP_20_-BSA) (LGC Biosearch Technologies). Splenocytes were loaded and cultured overnight in Dulbecco’s modified Eagle’s medium supplemented with 10% heat-inactivated fetal bovine serum, 1 mM sodium pyruvate, 10 mM *N*-2-hydroxyethylpiperazine-*N*′-2-ethanesulfonic acid, 1% non-essential amino acids, 100 U/ml penicillin, 100 μg/ml streptomycin, and 52 μM 2-mercaptoethanol. Then, individual wells were sequentially incubated with 1 μg/ml anti-IgG1 biotin (BD Biosciences, #A85–1) and streptavidin-conjugated horseradish peroxidase (Cytiva, 1:3000). Spots were visualized with insoluble tetramethylbenzidine (MOSS), and observed on an SMZ745 stereo microscope (Nikon Instech).

### Enzyme-linked immunosorbent assay (ELISA)

Serum was collected from tail vein or in some cases from heart under anesthesia with isoflurane. For sandwich ELISA, 96-well plates were coated with 2 μg/ml NP_20_-BSA. Serially diluted samples were loaded, followed by incubations with 1 μg/ml anti-IgG1 biotin (BD Biosciences, #A85–1) and streptavidin-conjugated horseradish peroxidase (Cytiva, 1:3000). Color development was performed with tetramethylbenzidine and stopped by acidification. Absorbance at 450 nm was measured on a 680 microplate reader (Bio-Rad Laboratories). Dose-response curves were analyzed, using R software (R Foundation for Statistical Computing) [22]. Antibody concentrations were determined, based on the 50% effective concentrations. N1G9 anti-NP IgG1 [23] was used as a standard.

### Flow cytometry

A single-cell suspension was prepared by passing tissues through a 100-μm nylon cell strainer. Red blood cell lysis was performed in lysis buffer (150 mM NH_4_Cl, 14 mM NaHCO_3_, and 2 mM EDTA). The following antibodies and reagents were used for flow cytometry: anti-CD19 allophycocyanin (APC)-Cy7 (BioLegend, #6D5, 0.25 μg/ml), anti-CD38 phycoerythrin (PE)-Dazzle594 (BioLegend, #90, 0.5 μg/ml), anti-CD45R (B220) PE-Cy7 (BioLegend, #RA3-6B2, 0.25 μg/ml), anti-CD95 APC (Miltenyi Biotec, #REA453, 0.15 μg/ml), anti-IgG1 fluorescein isothiocyanate (FITC) (BD Biosciences, #A85–1, 0.5 μg/ml), anti-Ig light chain κ (Igκ) Alexa Fluor 700 (BioLegend, #RMK-45, 1.25 μg/ml), NP_23_ PE (LGC Biosearch Technologies, 0.5 μg/ml), and propidium iodide (Sigma-Aldrich, 1 μg/ml). A flow cytometric analysis was performed on a Gallios cytometer (Beckman Coulter). Cell sorting was performed on a FACSAria II cytometer (BD Biosciences).

### Magnetic-activated cell sorting (MACS)

For enrichment of NP-specific non-GC B cells, B1–8 Ig heavy chain (IgH)-bearing cells pooled from three or more mice were incubated with a mixture of 1.25 μg/ml anti-CD11b biotin (Thermo Fisher Scientific, #M1/70), 1.25 μg/ml anti-CD43 biotin (BD Biosciences, #S7), 1.25 μg/ml anti-CD49b biotin (BioLegend, #DX5), 0.63 μg/ml anti-TER-119 biotin (BioLegend, #TER-119), and 1.25 μg/ml anti-Igκ biotin (BioLegend, #RMK-12), followed by negative selection with anti-biotin microbeads (Miltenyi Biotec), according to the manufacturer’s instructions. CD4^+^ T cells pooled from three or more mice were enriched using an IMag mouse CD4 T lymphocyte enrichment set (BD Biosciences). More than 75% purity was achieved, as measured by flow cytometry.

### Adoptive transfer

Donor cells were pooled from three or more mice per genotype. Adoptive transfer was performed by an IV injection of a mixture of sorted NP-specific IgG1^+^ memory B cells (2 × 10^3^ cells per recipient) and naïve splenocytes (1 × 10^7^ cells per recipient) or MACS-enriched CD4^+^ T cells (5 × 10^6^ cells per recipient). In some cases, naïve splenocytes alone were used as donor cells.

### In vitro cytokine production assays

According to the manufacture’s instructions, B cells were isolated from splenocytes with CD45R MicroBeads (Miltenyi Biotec). B220 positive B Cells (1.25 × 10^5^) were incubated in RPMI1640 supplemented with 10% fetal bovine serum, ﻿50 μM 2-mercaptoethanol, ﻿100 U/ml penicillin and 100 μg/ml streptomycin in the presence or absence of 20 μg/ml of ﻿anti-IgM F (ab′)_2_ antibody (Jackson ImmunoResearch Laboratories), or 10 μg/ml ﻿lipopolysaccharide (LPS; Sigma-Aldrich) for 72 h. Cytokines secreted in the supernatants were measured using LEGENDplex assay (BioLegend).

### Statistical analysis

Statistical analysis was performed using R software [24].

A *P* value of less than 0.05 was recognized to be significant.

## Supplementary Information

**Additional file 1: Supplemental Fig. 1** Gating strategies for NP-specific non-GC IgG1^+^ B cells (B220^+^CD19^+^CD38^+^CD95^−^NP^+^IgG1^+^). Prior to the analysis, Splenocytes were isolated from the mice described in Fig. 3. Numbers show percentages of cells in each gate.

**Additional file 2: Supplemental Fig. 2** Gating strategies for NP-specific non-GC IgG1^+^ B cells (B220^+^CD19^+^CD38^+^CD95^−^Igκ^−^NP^+^IgG1^+^). Prior to the analysis, B cells were isolated from B1–8 IgH-carrying or B1–8 IgH-carrying *Dock11* KO mice by MACS, as described in METHODS. Numbers show percentages of cells in each gate.

## Data Availability

The datasets used and/or analyzed during the current study are available within the article from the corresponding author on reasonable request.
