# Supplementary figures and images for: The immunosenescence-related factor DOCK11 is involved in secondary immune responses of B cells
Source: Immun Ageing. 2022 Jan 3;19:2. doi: 10.1186/s12979-021-00259-4 (PMC8722084; doi:10.1186/s12979-021-00259-4)

Figure S1

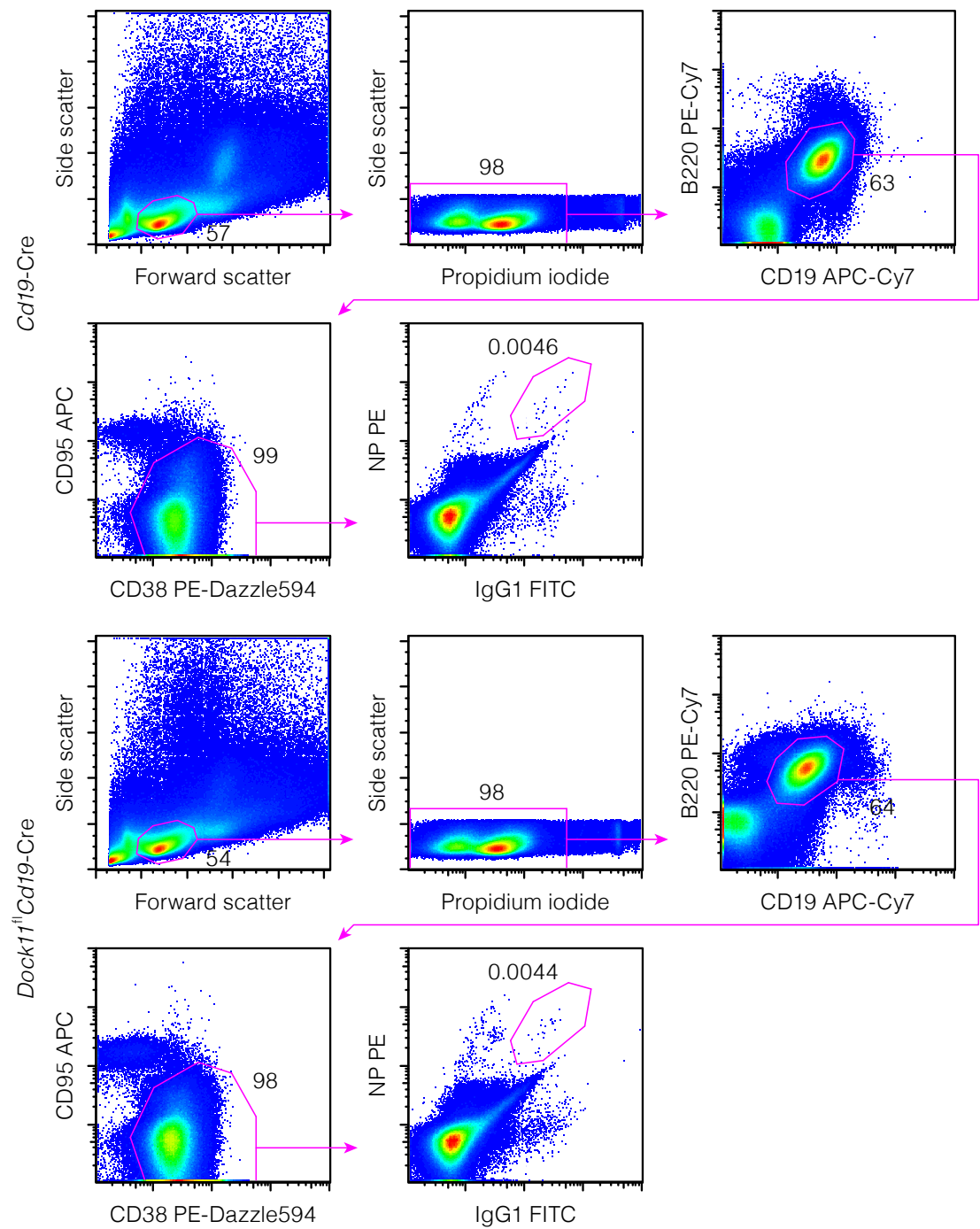

Supplement: Supplementary file 1 — Additional file 1: Supplemental Fig. 1 Gating strategies for NP-specific non-GC IgG1+ B cells (B220+CD19+CD38+CD95−NP+IgG1+). Prior to the analysis, Splenocytes were isolated from the mice described in Fig. 3. Numbers show percentages of cells in each gate. [file 12979_2021_259_MOESM1_ESM.pdf]

Figure S2

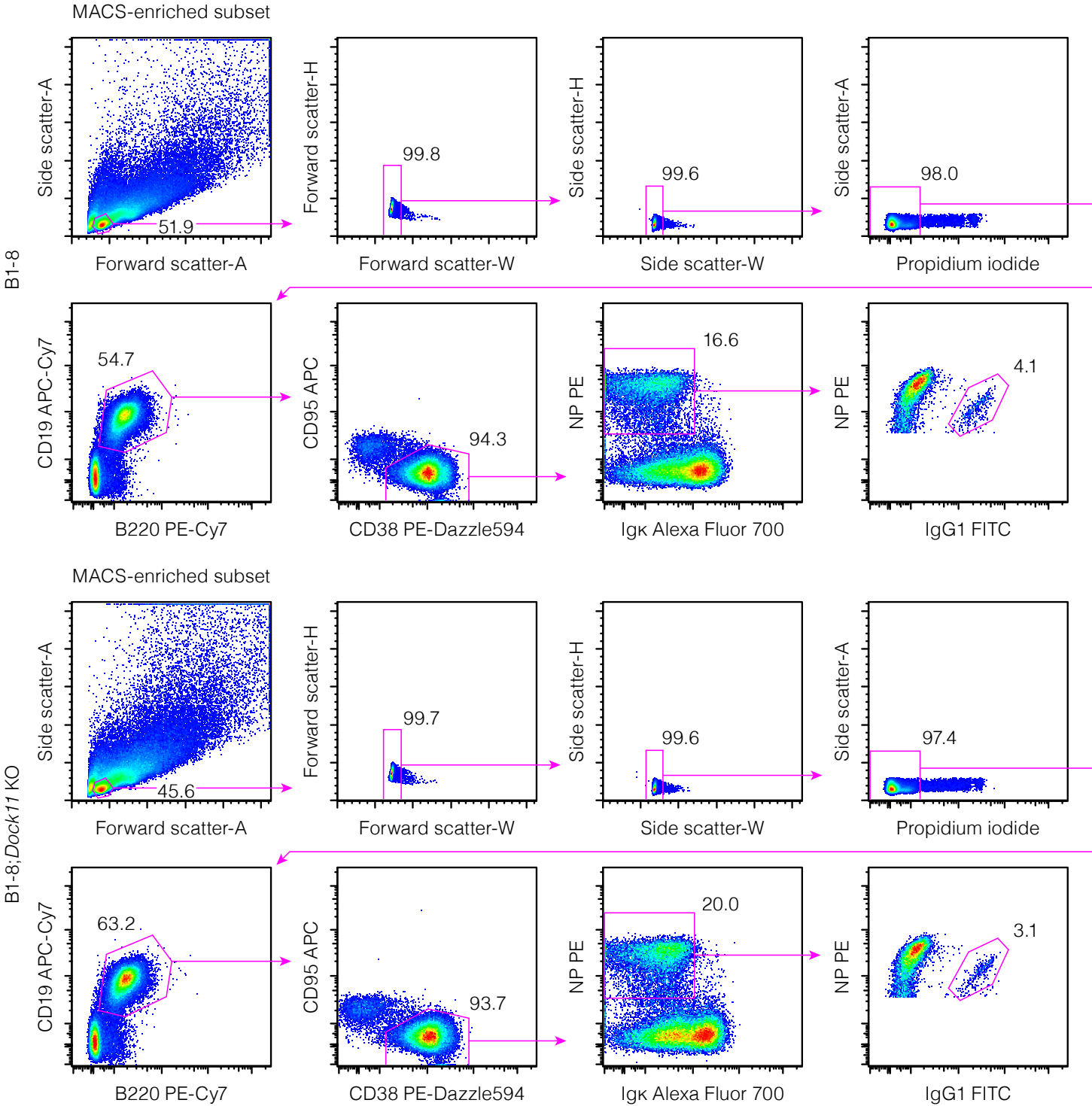

Supplement: Supplementary file 2 — Additional file 2: Supplemental Fig. 2 Gating strategies for NP-specific non-GC IgG1+ B cells (B220+CD19+CD38+CD95−Igκ−NP+IgG1+). Prior to the analysis, B cells were isolated from B1–8 IgH-carrying or B1–8 IgH-carrying Dock11 KO mice by MACS, as described in METHODS. Numbers show percentages of cells in each gate. [file 12979_2021_259_MOESM2_ESM.pdf]
